# Medicinal attributes of *Solanum xanthocarpum* fruit consumed by several tribal communities as food: an *in vitro* antioxidant, anticancer and anti HIV perspective

**DOI:** 10.1186/1472-6882-14-112

**Published:** 2014-03-28

**Authors:** Shashank Kumar, Abhay K Pandey

**Affiliations:** 1 Department of Biochemistry, University of Allahabad, Allahabad 211002, India

**Keywords:** Antioxidant, Anticancer, DPPH, LPOI, Reducing power, *Solanum xanthocarpum*, Anti HIV

## Abstract

**Background:**

*Solanum xanthocarpum* (Solanaceae) has been used for treatment of many infectious and degenerative diseases in traditional medicine. Present study reports the medicinal efficacy of *S. xanthocarpum* fruit as antioxidant, anticancer and anti HIV agents.

**Methods:**

Extracts were prepared using Soxhlet apparatus and partially characterized by thin layer chromatography (TLC). Total flavonoid content was determined spectrophotometrically. Reducing power, DPPH radical scavenging activity and lipid peroxidation inhibition assays were used for measurement of antioxidant potential. Cytotoxic (SRB assay) and anti-HIV RT inhibition (RT assay kit, Roche) activities were determined using ELISA.

**Results:**

TLC revealed the diversity of phytoconstituents in various sequential extracts of *S. xanthocarpum* fruit. Total flavonoid contents in extracts ranged between 10.22–162.49 μg quercetin equivalent/mg. Spectroscopic scanning of water soluble phenolics showed maximum absorbance at 250 and 280 nm. Polar extracts displayed potent radical scavenging activity (>80%). Several sub-fractions (spots) of extracts separated on TLC plates also exhibited powerful radical scavenging activity. Considerable reducing power was observed in extracts. Hexane fraction provided 55% lipoprotection in rat kidney homogenate. Non-polar extracts exhibited appreciable cytotoxic activity (70-91%) against leukemia (THP-1) and lung cancer (HOP-62) cell lines. Lower inhibitory activity was observed in extracts against HIV Reverse Transcriptase enzyme.

**Conclusion:**

The study demonstrated considerable antioxidant and anticancer activities in *S. xanthocarpum* fruit.

## Background

Excessive free radicals may produce oxidative stress that can damage lipids, proteins and DNA resulting into various chronic and degenerative diseases and/or disorders such as cancer, cardiovascular, alzheimer and ageing etc. [1]. Human body has several enzymatic and non-enzymatic antioxidant mechanisms to combat oxidative stress. The non enzymatic antioxidants are either produced naturally in the body or supplied through foods and/or supplements. Antioxidants derived from plants are presumed to be safe since they are natural in origin and have capability to counteract the damaging effect of reactive oxygen species (ROS) [2]. Phenolics are a group of naturally occurring compounds having functional hydroxyl groups. They have been reported to possess antioxidant and antiviral activities [3]. Free radical scavenging and inhibition of lipid peroxidation are two such mechanisms by virtue of which phenolics may combat the deleterious effect of ROS [4,5].

Oxidative stress induces a cellular redox imbalance which has been observed in various cancer cells. Polyphenols have been shown to inhibit the cancer associated enzyme telomerase, cell cycle and induce apoptosis [6]. Many important anticancer drugs are derived from plant sources, e.g., taxol from *Taxus brevifolia* and camptothecin from *Cascuta reflexa*[7]. Compounds having low side effects, inducing apoptosis and target specific cytotoxicity to the cancer cells are drugs of choice [8].

Uncontrolled viral replication in CD4 T cells of host body is responsible for pathogenesis of HIV. HIV reverse transcriptase (RT) enzyme is one of the prime targets for the treatment of HIV/AIDS. Inhibitors bind with the active or allosteric site of HIV RT based on their chemical nature whether they are analogs of nucleoside or non-nucleoside, respectively. This interaction may significantly reduce morbidity and mortality of HIV infected patients [9]. Several anti-HIV phytoconstituents of plant origin are known which include drymaritin (an alkaloid), diandraflavone, torosaflavone A, and cis-p-coumarate derived from a weed *Drymaria diandra*[10]. Phytoconstituents present in natural food items especially in edible fruits have been found to possess various pharmacological activities for example proanthocyanidin extracts of *Vitis vinifera* (Vitaceae) and ethanolic fraction of *Litchi chinensis (*Sapindaceae) fruits have been reported to exhibit potential antioxidant, anti carcinogenic and antiviral activities [11,12].

*Solanum xanthocarpum* Schrad. & Wendl (Solanaceae) is an annual herb which grows as wild plant in many parts of India. In vernacular it is known as Kantakari or Bhatkatiya. Fruits are berry, yellow or with white green strips, surrounded by enlarged calyx. Fruits are edible and local people of Manipur (India) use it as folk medicine for treatment of various ailments. Irula tribes of Hasanur Hills (Tamil Nadu, India) have history of consuming the cooked unripe fruits of *S. xanthocarpum* (Sx) as vegetable [13]. In Kerala, the Kattunaikka, Paniya and Kuruma tribes of Wayanad district consume fruits and seeds as food [14]. Fruits are considered as a valuable herbal product for traditional healers in treatment of many common diseases in other parts of India. In Ayurveda, medicinal use of Sx is well documented. Phytoconstituents present in Sx are used as anti-fertility, anti-inflammatory, anti-allergic agents and as potential fungicide [15,16]. Present manuscript reports the *in vitro* antioxidant, cytotoxic and anti-HIV activities of various polar and non-polar extracts of *S. xanthocarpum* fruit.

## Methods

### Plant material and preparation of extracts

*S. xanthocarpum* (Sx) fruits were collected from village Lalapur, Allahabad and were identified by experts in Botany Department, University of Allahabad, Allahabad, India. The voucher specimen has been kept in our department (AU/BCH/AKP/08). The fruits were shade-dried at room temperature and ground into fine powder. Powdered sample was sequentially extracted with different solvents i.e., hexane (HX), benzene (BZ), chloroform (CH), ethyl acetate (EA), acetone(AC), ethyl alcohol (ET) and water (AQ) in Soxhlet apparatus for 8 h [17,18]. The extract was centrifuged, filtered and dried under reduced pressure. The residues were dissolved in DMSO for assessment of biochemical activities of extracts.

### Thin layer chromatography (TLC)

TLC plates coated with silica gel G were prepared, dried and activated at 110°C for 90 min. The extracts were dissolved in respective solvents and spots were applied with the help of fine capillary tubes. Chloroform-ethyl acetate-formic acid (163:63:25) was used as the solvent system [19]. The phytoconstituents were visualized as bands after spraying with 10% H_2_SO_4_ and retardation factor (Rf value) was calculated. Water soluble phenolic contents were further identified as bluish bands after spraying with folin ciocalteau reagent (FCR 1:1 in water).

### DPPH radical scavenging assay on TLC plates

The radical scavenging assay was performed by the method of Cavin et al. [20]. The plate was sprayed with 0.2% 1,1-diphenyl-2-picrylhydrazyl (DPPH) methanolic solution followed by incubation in dark at room temperature for 30 min. The bands having free radical scavenging capability were identified as yellow spots against a purple background and Rf value was calculated.

### Spectroscopic scanning

The water soluble phenolic spots were eluted in respective solvents and scanned at different wave lengths (250, 280, 320, 370 and 510 nm) to identify presence of various phenolic groups of compounds such as isoflavones, flavanones, cinnamic acid, chalcones and flavones etc.

### Quantitative determination of total flavonoid content

Aluminum chloride colorimetric method of Chang et al. [21] as modified by us [16] was used for determination of flavonoid content. Small amount (0.2 ml) of different test extracts in DMSO (2 mg/ml) was taken followed by addition of methanol (1.8 ml), 10% aluminum chloride (0.1 ml), 1 M potassium acetate (0.1 ml) and distilled water (2.8 ml). Contents were mixed, incubated at room temperature for 30 min and then absorbance was measured at 415 nm. The calibration curve was prepared with quercetin (20–200 μg) and the flavonoid content in the test samples were expressed as μg quercetin equivalent/mg sample (μg QE/mg).

### Reducing power assay

The reducing power was determined by the method of Oyaizu [22] with slight modifications [3]. One ml extract (200–1000 μg/ml) in DMSO was taken. To each tube 2.5 ml of phosphate buffer (0.2 M, pH 6.6) and 2.5 ml of 1% potassium ferricyanide (K_3_Fe (CN)_6_) were added. Tubes were then incubated at 50°C for 20 min. The reaction was stopped by adding 2.5 ml of 10% TCA. One ml of the supernatant was mixed with 1 ml of distilled water and 0.5 ml of FeCl_3_ (0.1%, w/v) and kept at room temperature for 2 min. The absorbance was measured at 700 nm. Butylated hydroxytoluene (BHT) was used as positive control for comparison.

### DPPH radical scavenging assay

The free radical scavenging activity was measured by DPPH assay [23] as modified by us [2]. The modification included dissolution of extracts in DMSO instead of methanol. Three milliliter of 0.1 mM DPPH solution in methanol was added to 0.5 ml of the extracts (250, 500, 1000 μg/ml) dissolved in DMSO. The content was incubated at room temperature for 30 min in dark and absorbance was measured at 517 nm. The percentage scavenging activities (% inhibition) was calculated using the following formula

(1) $$\%\;I=\left[\left(\mathrm{Ac}–\mathrm{As}\right)/\mathrm{Ac}\right]\times 100$$

where I is inhibition, Ac and As are the absorbance of the control and the sample, respectively.

### Lipid peroxidation inhibition (LPOI) assay

The method described by Halliwell and Gutteridge [24] was followed to determine the amount of malondialdehyde (MDA) formation with slight modifications [25]. Rat kidney homogenate (10% w/v) was prepared in phosphate buffer (0.1 M, pH 7.4 having 0.15 M KCl). The protective effect of Sx fruit fractions was determined by taking 100 μl extract solutions (2 μg/μl) prepared in respective solvents and evaporated to dryness followed by addition of 1 ml KCl (0.15 M) and 0.5 ml of tissue homogenate. Peroxidation was initiated by adding 100 μl ferric chloride (10 mM). After incubation at 37°C for 30 min thiobarbituric acid reactive substances (TBARS) were estimated by adding 2 ml of ice-cold HCl (0.25 N) containing 15% TCA, 0.5% TBA and 0.5% BHT to the reaction mixture, followed by heating at 100°C for 60 min. The tubes were cooled and centrifuged. The absorbance of the supernatant was measured at 532 nm. The percent LPOI (%I) was calculated by the equation no. 1 given above.

### Anti-HIV activity

The HIV-RT inhibition was performed by using an RT assay kit (Roche) [26]. The percentage inhibitory activity of RT inhibitors (extracts) was calculated by comparing with a sample that did not contain an inhibitor using following formula:

(2) $$\%\;\mathrm{Inhibition}=100-\left[\left(A_{\mathrm{WI}}/A_{\mathrm{WOI}}\right)\times 100\right]$$

Where *A*_WI_ is absorbance of control and *A*_WOI_ is absorbance of sample solution.

### Cell lines and growth conditions

Human cancer cell lines namely, lungs (HOP-62) and leukemia **(**THP-1) cell lines were procured from National Center for Cell Sciences, Pune, India. Cell lines were grown and maintained in RPMI-1640 medium, pH 7.4 with 10% FCS, 100 units/ml penicillin, 100 μg/ml streptomycin and 2 mM glutamine. Cells were grown in CO_2_ incubator (Heraeus, GmbH Germany) at 37°C in the presence of 90% humidity and 5% CO_2_.

### Cytotoxic assay by sulforhodamine B dye (SRB assay)

The *in vitro* cytotoxicity of fruit extracts was determined using sulforhodamine-B (SRB) assay [27]. Cell suspension (100 μl) was incubated for 24 h. 100 μl test extract in DMSO (100 μg/well) was then added to the wells and cells were further incubated for another 48 h. The cell growth was arrested by layering 50 μl of 50% TCA and incubated at 4°C for an hour followed by washing with distilled water and air-dried. SRB (100 μl, 0.4% in 1% acetic acid) was added to each well and plates were incubated at room temperature for 30 min. The unbound SRB dye was washed with 1% acetic acid and plates were air dried. Bound dye was dissolved in Tris–HCl buffer (100 μl, 0.01 M, pH 10.4) and the absorbance was recorded on ELISA reader at 540 nm. Suitable blanks and positive controls were also included.

### Statistical analysis

All experiments were carried out in triplicate and data were expressed as mean ± standard deviation (SD) or standard error of mean (SEM). The plots were prepared using Microsoft excel and Graph pad Prism software. Data were analyzed using One-way and Two-way ANOVA and the values of *p* < 0.001 were considered as statistically significant.

## Results

### Thin layer chromatography of extracts

**Figure 1:**
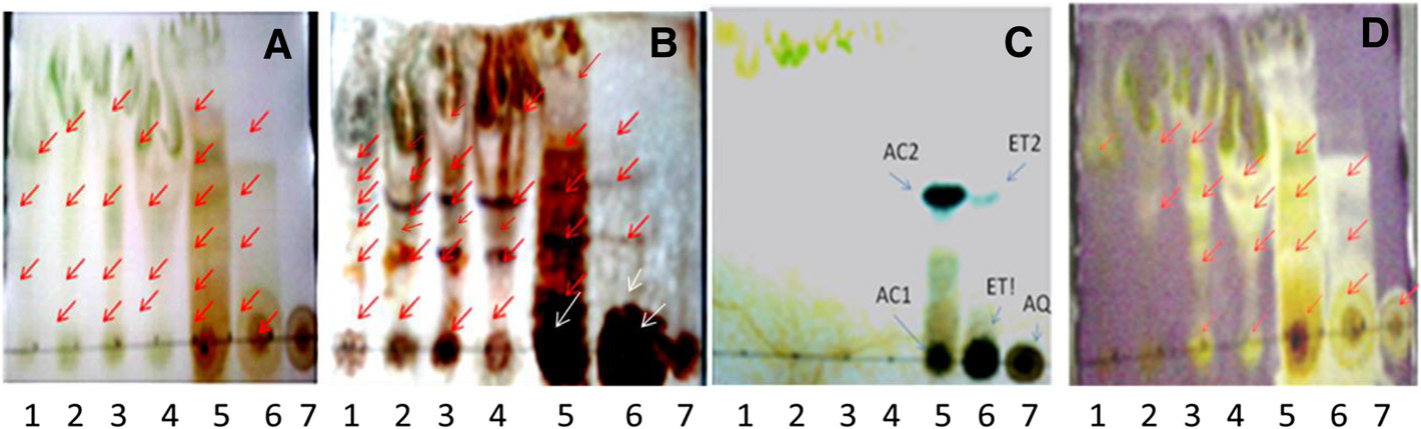
**Thin layer chromatogram of *S. xanthocarpum *fruit extracts. (A)** Untreated, **(B)** Treated with 10% H_2_SO_4_, **(C)** Water soluble phenolics **(D)** Plate assay for DPPH radical scavenging activity of separated components. Numbers 1–7 indicate hexane, benzene, chloroform, ethyl acetate, acetone, ethyl alcohol and water extracts, respectively.

Separation of components is shown in Figure 1A and B. The Rf values of spots are given in Table 1. Number of spots (Rf 0.03-0.77) on chromatogram became visible after treatment with 10% H_2_SO_4_. Many non-polar fractions exhibited bands having similar mobility (Rf 0.22 and 0.42). Polar fractions (AC and ET) also revealed components having similar Rf value (0.27). FCR treatment exhibited presence of water soluble phenolics in AC, ET and AQ extracts (Figure 1C and Table 1).

**Table 1:** **Total flavonoid content in *S. xanthocarpum *** **fruit extracts and Rf value of spots on chromatogram**

**Extracts**	**Flavonoids (μg QE/mg)**	**Rf values**							
HX	71.82 ± 0.08	0.05^b^	0.22^a^	0.35^b^	0.42^a^	0.50^b^	0.59^ad^	-	-
BZ	69.76 ± 0.12	0.05^a^	0.22^a^	0.35^b^	0.42^ad^	0.51^b^	0.64^ad^	-	-
CH	59.51 ± 0.13	0.03^bd^	0.05^a^	0.22^ad^	0.35^b^	0.42^ad^	0.53^b^	0.70^ad^	-
EA	162.49 ± 0.15	0.06^ad^	0.22^ad^	0.35^b^	0.42^ad^	0.62^bd^	0.71^a^	-	-
AC	148.07 ± 0.18	0.05^acd^	0.14^a^	0.27^ad^	0.31^b^	0.44^ad^	0.57^acd^	0.69^a^	0.77^b^
ET	71.43 ± 0.14	0.05^acd^	0.14^b^	0.27^ad^	0.45^ad^	0.58^acd^	-	-	-
AQ	10.22 ± 0.12	0.00^acd^	-	-	-	-	-	-	-

*Abbreviations*: *HX* hexane, *BZ* benzene, *CH* chloroform, *EA* ethyl acetate, *AC* acetone, *ET* ethyl alcohol, *AQ* water.

Values with superscript ‘^a^’ indicates spots on the untreated chromatogram; ‘^b^’ indicates additional spots visualized after 10% H_2_SO_4_ treatment; ‘^c^’ indicates water soluble phenolics after treatment with FCR reagent (1:1 in water); and ‘^d^’ denotes phytoconstituent spots having DPPH radical scavenging ability.

Many phytoconstituent spots on TLC plates exhibited appreciable DPPH radical scavenging activity (Figure 1D) and their Rf values are shown in Table 1.

### Spectroscopic scanning

Spectroscopic scanning data of water soluble phenolic spots eluted from TLC chromatogram (Figure 1C) are shown in Table 2. AQ and AC1 spots exhibited maximum absorbance at 280 nm while rest of the spots (ET1, ET2 and AC2) showed absorption maxima at 250 nm. None of the fractions demonstrated absorbance at 510 nm.

**Table 2:** **Spectroscopic scanning data of water soluble phenolic spots obtained from thin layer chromatogram of *S. xanthocarpum *** **fruit extracts**

**Wavelength (nm)**	**Absorbance**				
	**AQ**	**ET**_**1**_	**ET**_**2**_	**AC**_**1**_	**AC**_**2**_
250	0.1880	4.0000	3.5042	-	4.0000
280	0.2897	-	0.0851	4.0000	-
320	0.0309	1.0752	0.8752	0.6494	0.5701
370	-	-	0.5432	0.3420	0.4990
510	-	-	-	-	-

*Abbreviations*: *AQ* water, *ET* ethyl alcohol, *AC* acetone exracts.

### Yield of the extracts and total flavonoid content

The yield of *S. xanthocarpum* fruit extracts increased with polarity of the solvents used for extraction in the order HX (0.1%), BZ (0.2%), CH (0.4%), EA (7%), AC (5%), ET (12%) and AQ (38%). Extracts were quantified for total flavonoid contents (Table 1). EA extract accounted for maximum flavonoid content (162.49 ± 0.15 μg QE/mg) followed by AC extract (148.07 ± 0.18 μg QE/mg). The order of flavonoid content in extracts was EA, AC, HX, ET, BZ, CH and AQ.

### Reducing power of extracts

**Figure 2:**
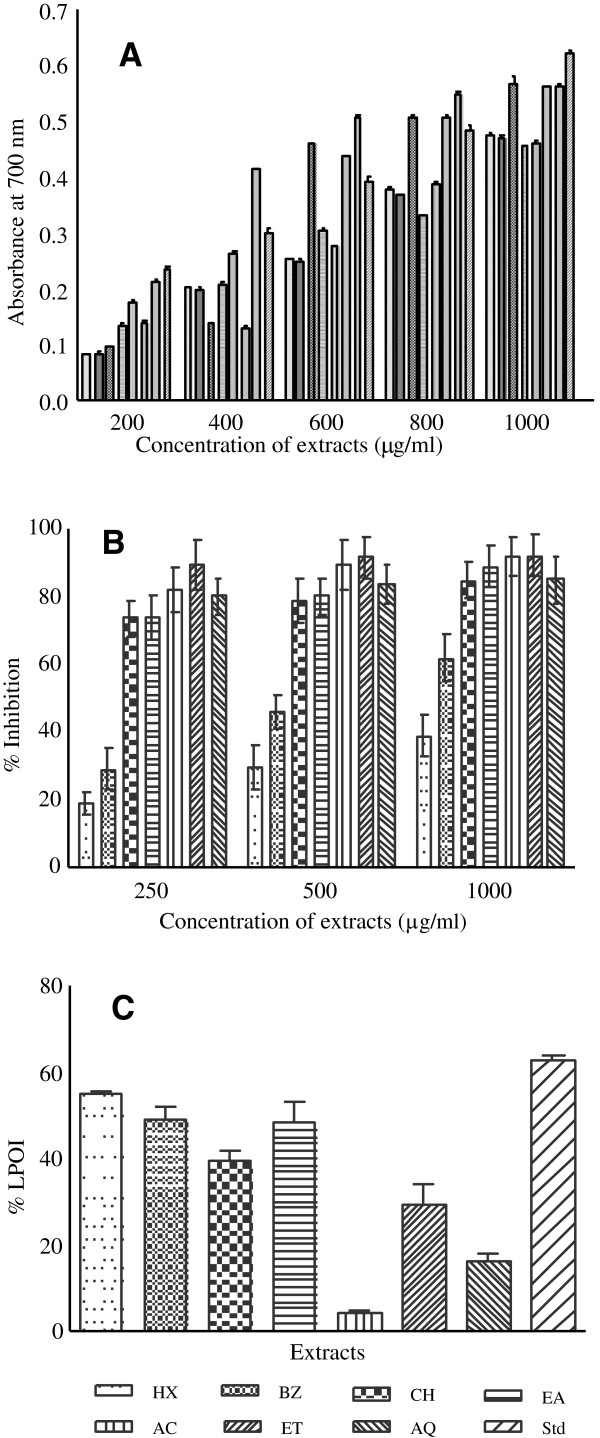
**Antioxidant capacity assessment of *S. xanthocarpum *fruit extracts. (A)** Reducing power ability of extracts, **(B)** DPPH radical scavenging activity, **(C)** Protective activity of extracts against lipid peroxidation. Extracts were prepared in HX-hexane, BZ-benzene, CH-chloroform, EA-ethyl acetate, AC-acetone, ET-ethyl alcohol and AQ-water as described in Methods section. Standard antioxidant compounds (Std) were used as control for comparison. Reducing power was measured at different concentration of extracts (200–1000 μg/ml) and BHT was used as control. Radical scavenging activity of extracts was evaluated in the concentration range 250–1000 μg/ml. Lipid peroxidation inhibition (%LPOI) activity was determined in presence of 200 μg of extract in reaction mixture and BHA was used as standard. The results are expressed as mean ± SD (n = 3). Antioxidant activities of the extracts varied significantly with different concentrations (p < 0.001).

Concentration dependent (200–1000 μg/ml) reducing ability was observed (Figure 2A). At higher concentration the reducing activities of CH, ET and AQ extracts was 0.568, 0.559 and 0.563, respectively. The reducing power of potential Sx fruit extracts was comparable to the activity shown by BHT.

### DPPH radical scavenging activity

Free radical scavenging activity of Sx fruit extracts are shown in Figure 2B. Most of the extracts demonstrated appreciable radical scavenging potential (about 80%) even at lowest test concentration (250 μg/ml). Further increase in concentration led to saturation effect (74-92%). Standard antioxidants namely BHA, BHT, quercetin and ascorbic acid have already been reported to produce 95-99% scavenging activities [2].

### Protective effect of extracts against lipid peroxidation

Lipo protective efficacy (% LPOI) of extracts against per-oxidative damage in tissue homogenate is shown in Figure 2C. Non-polar fractions exhibited 48-55% protection against peroxidation while other fractions accounted for less than 30% activity. Butylated hydroxy anisole (BHA) displayed about 63% protection against membrane damage.

### Anti-HIV activity

**Figure 3:**
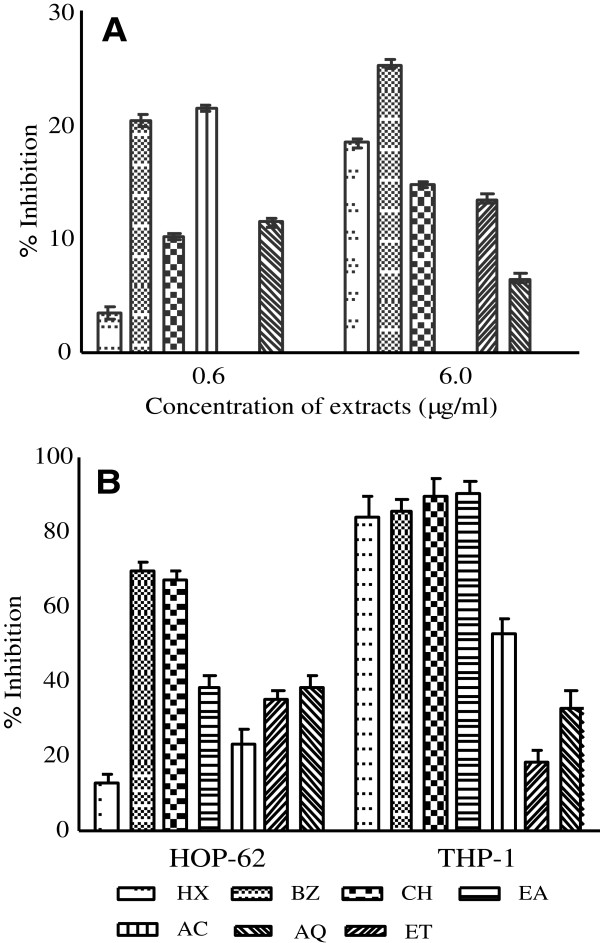
**Anti-HIV and Cytotoxic activities of *S. xanthocarpum* fruit extracts. (A)** Anti-HIV reverse transcriptase (RT) activity was measured at extract concentrations of 0.6 and 6.0 μg/ml. **(B)** Cytotoxic potential of extracts was measured against HOP-62 (lung) and THP-1 (leukemia) human cancer cell lines in presence of 100 μg of extract per well. The results are expressed as mean ± SD of three replicates. Anti-HIV and cytotoxic activity of *S. xanthocarpum* fruit varied significantly with different extracts (p < 0.001). Abbreviations: HX-hexane, BZ-benzene, CH-chloroform, EA-ethyl acetate, AC-acetone, ET-ethyl alcohol and AQ-water extracts.

Sx fruit extracts were monitored for anti-RT activity at different concentrations (0.6 and 6.0 μg/ml). Non polar extracts showed dose dependent inhibitory activity. The anti-HIV RT activity in general was low as shown in Figure 3A. Nevirapine, the standard anti-HIV drug (not shown in figure) demonstrated 99.67% inhibitory activity.

### Cytotoxic activity

The cytotoxic activity of extracts against HOP-62 (lung) and THP-1 (leukemia) are depicted in Figure 3B. Non polar extracts (HX, BZ, CH and EA) exhibited considerable anticancer potential showing 85-91% growth inhibition of THP-1 cell line while BZ and CH fractions accounted for about 70% cytotoxicity against lung cancer cell line (HOP-62). Polar extracts in general displayed lower cytotoxic activity.

## Discussion

Plants are natural repositories of molecules with diverse structure and function. Many phytoconstituents exhibit nutritive and pharmacological activities [28-30]. In the current study compounds present in sequential extracts of *S. xanthocarpum* fruit were screened for their biochemical potential such as antioxidant, cytotoxic and anti-HIV activities. Thin layer chromatography enables the fractionation and identification of secondary compounds (Figure 1, Table 1). AC fraction showed maximum variability of phyto-constituents as indicated by number of spots. In non polar fractions (HX and CH) more spots became visible after treatment with H_2_SO_4_. It could be inferred that phytoconstituents in HX and CH fractions might have absorption in UV range. A single wavelength is not enough to study mixture of phenolic compounds because different polyphenols show absorption at specific wave lengths. Isoflavones and benzoic acids absorb at 250 nm; flavanones, catechins, ellagic acid and flavones at 280 nm; cinnamic acids, flavones and chalcones at 320 nm; flavonols at 370 nm and anthocyanins absorb at 510 nm [31-33]. Spectroscopic scanning of water soluble phenolic spots (AC, ET and AQ) showed maximum absorbance at 250 and 280 nm (Figure 1C, Table 2). Absorption maxima for ET1, ET2 and AC2 spots at 250 nm indicates the presence of isoflavones and/or benzoic acid while AQ and AC1 absorbed maximally at 280 nm indicating presence of flavanones, flavones, catechins and/or ellagic acid. Besides absorption at 250 and 280 nm, all the water soluble phenolic spots also showed absorbance at 320 nm indicating presence of cinnamic acid, chalcones and flavones in the extracts (Table 2).

EA and AC extracts of fruit were found to have noticeable quantity of flavonoid content (Table 1). Chemically flavonoids are diphenylpropanes and more than 4000 flavonoids have been isolated from plants [33]. Many biological activities such as lipoprotective, anti platelet, and anti inflammatory are related to the anti-oxidative effects of flavonoids [16]. The mechanism behind antioxidant property of flavonoids includes radical scavenging, reducing ability, metal ion chelation and inhibition of enzymatic systems responsible for free radical generation [33].

**Figure 4:**
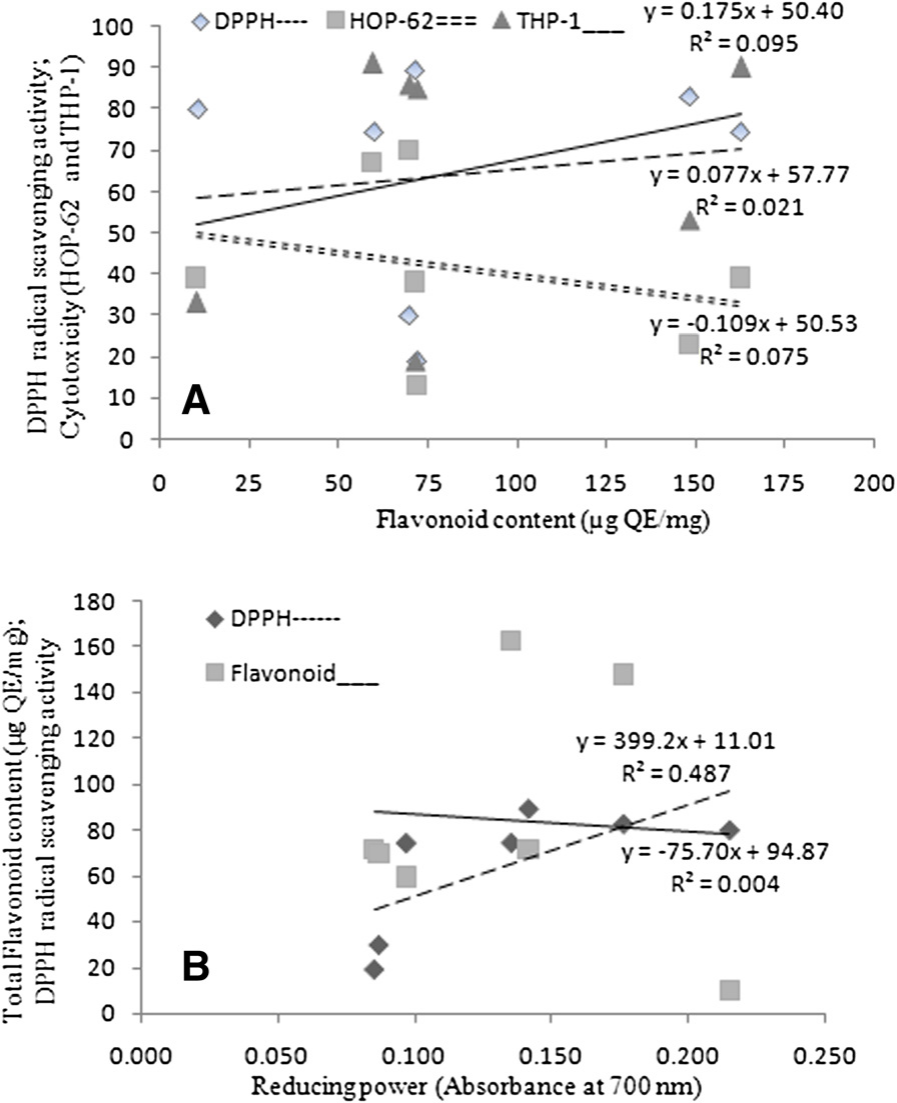
**Correlation of flavonoids with *in vitro *biochemical activities. (A)** Relationship of total flavonoid content with DPPH radical scavenging activity and cytotoxic activity, **(B)** Relationship of reducing power with total flavonoid content and DPPH radical scavenging activity of *S. xanthocarpum* fruit extracts.

Violet colour of DPPH, the commercially available stable free radical, is reduced to a pale yellow colour due to the abstraction of hydrogen atom from antioxidant compound. Appearance of yellow coloured spots against purple background in TLC-DPPH plate assay indicated potential antioxidant activity in many sub fractions of EA, AC and ET extracts as well as in AQ sample (Figure 1D, Table 1). The intensity of the yellow colour depends on the quantity and the nature of the compounds present at that location [20]. In quantitative DPPH radical scavenging assay most of the Sx fruit extracts except HX and BZ demonstrated appreciable radical scavenging activity at 250 μg/ml concentration revealing considerable antioxidant potential in the extracts (Figure 2B). Our findings are corroborated by the reports on other plants that DPPH radical scavenging activity of extracts is mediated by their hydrogen donating ability [34,35]. Both the assays i.e., qualitative (DPPH-TLC) and quantitative (DPPH) assays substantiate the prospective use of *S. xanthocarpum* fruit as a food having potent antioxidant activity. A weak positive correlation between flavonoid content and DPPH radical scavenging activity was observed (Figure 4A). Prasad et al. [36] have also reported a weak correlation between flavonoid content and the radical scavenging activity.

Reducing power of plant extracts is generally associated with the presence of reductones, which exert antioxidant action by breaking the free radical chain through donating a hydrogen atom [37]. In this assay, Fe^3+^/ferricyanide complex is reduced to the blue colored ferrous form by antioxidants with absorption maxima at 700 nm [37]. Some of the extracts in present study exhibited considerable reducing power at higher concentrations indicating their hydrogen donating capacity (Figure 2A). A negative correlation was found between flavonoid content and the reducing power of the extracts (Figure 4B) which is supported by the report of Liu et al. [38]. However a positive correlation between reducing power and radical scavenging ability of the extracts was found (Figure 4B). Since both the properties are due to hydrogen donating ability of the test sample it might be concluded that in addition to flavonoids there are other phytochemical moieties responsible for the antioxidant potential of *S. xanthocarpum* fruit extracts [39].

Redox chemistry of iron plays an important role in the occurrence and the rate of lipid peroxidation. Fe^3+^ reacts with lipid hydroperoxides to form peroxyl radicals which ultimately results in malondialdehyde (MDA) formation. MDA is usually taken as a marker of LPO and oxidative stress [40]. Some of the non-polar extracts accounted for about 40-55% lipo-protective activities (Figure 2C). This revealed that major phytoconstituents present in the active extracts are responsible for quenching Fe^3+^ and thereby preventing oxidative damage to lipids and in turn protecting the tissues [24,25]. Phenolics have been shown to delay or prevent the progression of various diseases by averting peroxidation of membrane lipids [8,35].

ROS can cause DNA damage and has been implicated in carcinogenesis [41]. *S. xanthocarpum* extracts showed potent cytotoxic activities against leukemia cell line (THP-1). However lower activity was observed against lung cancer cell line (HOP-62). This may be attributed to the fact that different cell lines might exhibit different sensitivities to cytotoxic agents [42]. The results revealed a positive correlation between flavonoid content and THP-1 cell line growth inhibition (Figure 4A). Similar relationship between flavonoids and cytotoxic activities has also been reported in other studies [43]. Flavonoids acting as antioxidants have been reported to inhibit carcinogenesis. The position, number, and substitution of the hydroxyl of the rings in flavonoid may be important factors affecting their cytotoxic activities [33]. Some of the flavonoids such as apigenin, fisetin, quercetin and luteolin are known to be potent inhibitors of cancer cell proliferation [7,39,44]. There are several mechanisms of the cytotoxicity of flavonoids, including the inhibition of topoisomerases and kinases [33].

Natural products have been shown to inhibit various stages of the replication cycle of the HIV [45]. Many viruses reported to be affected by phytochemicals include herpes simplex virus, respiratory syncytial virus, parainfluenza virus, and adenovirus [46]. Phytoconstituents have potential to exhibit both anti infective and anti replicative abilities. *S. xanthocarpum* fruit extracts in our study exhibited low inhibitory activity against HIV RT. Since numerous chemical moieties (with or without activity) are present in crude extract, it might be possible that isolation and purification of the active ingredients from potential extracts may provide enhancement in anti-HIV activity.

## Conclusion

The present study revealed that the phytoconstituents present in *S. xanthocarpum* fruit possess considerable antioxidant and cytotoxic potential. They are potent scavengers of free radicals and have reducing ability. Therefore they prevent ROS mediated lipid damage. The study validated the use of *S. xanthocarpum* fruit by tribal communities in traditional medicine. Isolation and characterization of specific chemical moieties having potential biologic activities may provide an effective antioxidant and anticancer agents in future.

## Competing interests

Authors declare that they do not have any conflict of interests.

## Authors’ contribution

AKP participated in the research design, analysis of the data and drafting of the manuscript. SK conducted all the experiments. Both the authors have read and approved the final manuscript.

## Pre-publication history

The pre-publication history for this paper can be accessed here:

http://www.biomedcentral.com/1472-6882/14/112/prepub
